# Recurrent Fanconi Syndrome Induced by Lenalidomide: A Case Report

**DOI:** 10.7759/cureus.66118

**Published:** 2024-08-04

**Authors:** Irida Sharra, Marine Lhoir, Jean-Marin Des Grottes, Melanie Vaes, Fabien Guerisse

**Affiliations:** 1 Emergency Department, CHU Charleroi-Chimay, Charleroi, BEL; 2 Nephrology Department, CHU Tivoli, La Louvière, BEL; 3 Hematology Department, Chu Tivoli, La Louvière, BEL

**Keywords:** lenalidomide, multiple myeloma, drug toxicity, proximal renal tubule, fanconi syndrome

## Abstract

Fanconi syndrome (FS) is a complex disorder characterized by a reabsorption defect in the proximal renal tubule (PT), leading to urinary loss of molecules such as glucose, phosphate, calcium, amino acids, bicarbonate, potassium, and low-molecular-weight proteins. Its etiology can be genetic or acquired, with drug toxicity being a significant cause of the acquired forms. The heterogeneous manifestations of FS, whether in its partial or complete form, can pose challenges in the emergency department; nevertheless, it should be considered in certain patients, as understanding its cause is crucial for initiating effective treatment.

We present the case of a 59-year-old female patient with FS who was treated with lenalidomide in the context of stage III IgG kappa multiple myeloma according to the Salmon Durie classification. We highlight the recurrent nature of this syndrome in this patient.

## Introduction

Fanconi syndrome (FS) is characterized by dysfunction within the proximal renal tubule and its transporters, leading to the loss of molecules such as glucose, phosphate, calcium, amino acids, bicarbonate, and potassium. This widespread defect contrasts with specific tubulopathies, where often only one transporter or receptor is involved [1,2]. Its etiology can be genetic, with autosomal recessive transmission (1 in 40,000 births) or acquired. In the latter group, the most common causes are drugs, toxic substances (such as heavy metals), or monoclonal immunoglobulin deposition disease [3,4].

This article reports the case of a patient admitted to the emergency department who presented a recurrence of FS during a new cycle of lenalidomide. To date, only a few cases have been described [5,6], making the recurrent nature of this patient particularly noteworthy.

## Case presentation

A 59-year-old female patient was admitted to the emergency department for severe hypokalemia (1.7 mmol/L). Her history revealed asthenia, xerostomia, and hyposalivation associated with odynophagia. Her medical history included stage III IgG kappa multiple myeloma according to the Salmon Durie classification and an Injury Severity Score (ISS) II score, diagnosed in 2012. She underwent an autologous stem cell transplant in February 2018 followed by maintenance treatment with cycles of lenalidomide starting in April 2018, with the last cycle 28 days before admission. She had previously experienced the first episode of severe hypokalemia a few months earlier, but no further investigations had been conducted. Additionally, she had type II non-insulin-dependent diabetes, obesity, hypercholesterolemia, and untreated arterial hypertension. The patient had no known dependencies on alcohol or tobacco and no known allergies. Her medication regimen included tinzaparin 13,000 IU once daily, atorvastatin 40 mg once daily, pantoprazole 40 mg once daily, calcium carbonate 1g once daily, D-cure once weekly, and metformin 850 mg once daily. This treatment regimen had not been modified before admission to the emergency department.

**Figure 1:**
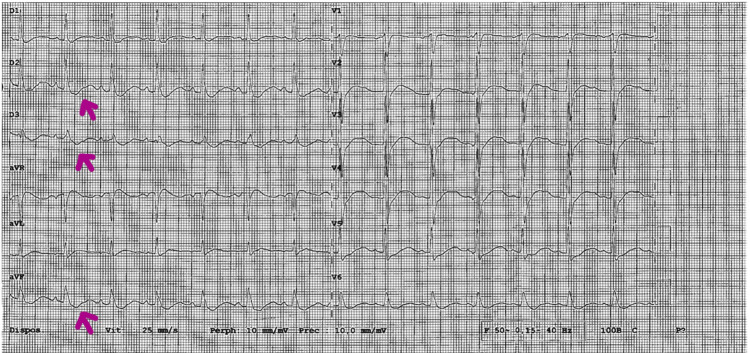
ECG at the admission in the ER revealing severe repolarization disorder (lying italic S wave) ECG: Electrocardiogram; ER: emergency department

The clinical examination was unremarkable except for asthenia and overweight. The electrocardiogram showed a widened QRS complex at 0.14 seconds (N = 0.07-0.11 sec) with severe repolarization disorder (Figure 1). 

Laboratory tests revealed impaired renal function (estimated glomerular filtration rate (eGFR)-Chronic Kidney Disease Epidemiology Collaboration (CKD-EPI): 31 mL/min/1.73m2; N > 60 mL/min/1.73m2), associated with severe hypokalemia (1.7 mmol/L; N = 3.5-5.1 mmol/L), hypophosphatemia (0.22 mmol/L; N = 0.8-1.45 mmol/L), hypochloremia (94 mmol/L; N = 98-107 mmol/L), and normal sodium levels (137 mmol/L; N = 136-145 mmol/L). Notably, there was an inflammatory syndrome (C-reactive protein (CRP) at 122 mg/L; N < 5 mg/L) without associated leukocytosis, thrombocytopenia (52,000 platelets/mm³; N = 150-410/mm³), and hyperglycemia at 184 mg/dL (N = 70-110 mg/dL). Urinalysis revealed significant glycosuria (579.7 mg/dL; N < 15 mg/dL), urinary loss of potassium (excretion fraction: 18.2%), and phosphaturia (excretion fraction: 40.8%), as well as microalbuminuria (210 mg/L; N = 0.01-30 mg/L) and hypoosmolarity (175 mosm/kg). 

Due to the ECG abnormalities and biological alterations, the patient was transferred to the intensive care unit. The diagnosis of acquired FS was considered, and lenalidomide was discontinued. Following intravenous supplementation, the electrolyte disorders were corrected, and the ECG normalized, allowing the patient to be discharged from the hospital eight days after her admission.

## Discussion

Acquired FS was considered, characterized by glycosuria, hypokalemia with urinary potassium loss (excretion fraction: 18.2%), and hypophosphatemia (excretion fraction: 40.8%; normal < 20%) [7]. 

The most likely etiology is drug toxicity, particularly from lenalidomide. Indeed, metabolic disorders in this patient, with a history of multiple myeloma for several years, occurred in the months following the start of carfilzomib treatment (which has not been described among the side effects) and lenalidomide, thus establishing a temporal relationship (Table I). 

**Table 1:** Comparison of metabolic disorders in our patient

Presentation of Fanconi Syndrome	First Episode	Second Episode
	June 25, 2018	December 24, 2018
Start date of lenalidomide cycles	April 30, 2018	July 10, 2018
Serum potassium	2.6 mmol/L	1.7 mmol/L
Serum sodium	141 mmol/L	137 mmol/L
Serum phosphate	0.44 mmol/L	0.22 mmol/L
Serum creatinine	0.76 mg/dL	1.43 mg/dL
pH	Not measured	7.54
**Urinalysis:**		
-Glucose (glycosuria)	Not measured	579.97 mg/dL
-Urinary potassium	Not measured	5 mmol/L
-Urinary phosphate	Not measured	<1.45 mmol/L
-Urinary sodium	Not measured	7.1 mmol/L
-Urinary creatinine	Not measured	23.1 mg/dL
-Amino acid excretion (amino-aciduria)	Not measured	Not measured
-Urinary osmolarity	Not measured	175 mOsm/kg (normal range: 300-900 mOsm/kg)

Moreover, following the discontinuation of treatment, the patient no longer presented with electrolyte disorder. For these reasons, other etiologies such as tubular damage due to monoclonal gammopathy seem less plausible.

A question that could be raised is the discontinuation of treatment after the first episode of FS. This is debatable because lenalidomide is an important drug for multiple myeloma treatment. Therefore, the risk-benefit balance should be assessed on a case-by-case basis.

Lenalidomide is an immunomodulatory drug analogue to thalidomide with multiple mechanisms of action. It has an antiproliferative effect through the reduction of vascular endothelial growth factor (VEGF) and interleukin 6 (IL-6) expression. Lenalidomide also has immunomodulatory properties by increasing the activity of T-cells, CD4, and CD8 [8] and a direct antitumor effect as a cytotoxic drug, thus inducing apoptosis [9-11].

As noted by Hall et al., the incidence of drug-induced FS is probably underestimated because there are few systematic studies, and the markers usually used, such as eGFR, serum creatinine, and the urinary albumin/creatinine ratio, are not sensitive for detecting dysfunction of the proximal renal tubule (PT) [1]. The PT is the main site of excretion for several drugs and the most common site for drug-induced toxicity. In FS, the dysfunction of several transporters at the level of the PT involves the loss of molecules that are mainly reabsorbed at this level. For others like sodium, potassium, chloride, magnesium, and calcium, alternative reabsorption mechanisms, especially in the distal tubule, are involved. This explains why their loss may not be significant [1].

The mechanisms of transporter dysfunction remain unclear, but it seems that mitochondrial alteration may be involved through the blocking of PT transporters [1,2].

The clinical characteristics of FS are well outlined in the existing literature. In partial FS, only some abnormalities are present. Generalized muscle weakness, polydipsia, and polyuria may be observed, as well as specific clinical presentations in cases of metabolic acidosis and/or hypokalemia [1,3,4], which are not always constant due to compensatory mechanisms. The main characteristic remains hypophosphatemia, which can lead to bone demineralization.

It is essential to investigate a temporal relationship [1], despite reports of toxicity manifesting months or even years subsequently [12]. More sensitive markers to detect PT dysfunction should be used, such as urinary beta2-microglobulin and retinol-binding protein (RBP) [13,14]. Indeed, a dysfunction of PT is not always accompanied by glomerular dysfunction. Consequently, the tests commonly used to monitor renal function, such as the eGFR based on serum creatinine, may not be altered [15]. 

The goal is twofold: firstly, the correction of biological abnormalities, and secondly, the withdrawal of the implicated drug. Although cases evolving toward chronic renal failure exist, in most cases, drug-induced FS is reversible upon discontinuation of treatment [2].

## Conclusions

We have described a case of recurrent FS secondary to lenalidomide toxicity, which remains poorly documented in the literature. The purpose of our case report is to emphasize the importance of early detection of PT dysfunction in at-risk patients, such as oncology patients or those with underlying renal insufficiency, particularly when initial electrolyte disturbances, such as hypophosphatemia, appear. Consequently, additional urinary tests should be included to confirm or rule out FS. Furthermore, we aim to highlight how a commonly used medication in multiple myeloma, such as lenalidomide, can cause rare and potentially irreversible damage.
